# Two-Dimensional Metal–Organic Framework on Superconducting NbSe_2_

**DOI:** 10.1021/acsnano.1c05986

**Published:** 2021-11-03

**Authors:** Linghao Yan, Orlando J. Silveira, Benjamin Alldritt, Shawulienu Kezilebieke, Adam S. Foster, Peter Liljeroth

**Affiliations:** † Department of Applied Physics, Aalto University, 00076 Aalto, Finland; ‡ Nano Life Science Institute (WPI-NanoLSI), Kanazawa University, Kakuma-machi, Kanazawa 920-1192, Japan

**Keywords:** electronic structures, metal−organic framework, scanning tunneling microscopy, tunneling spectroscopy, on-surface synthesis, 2D material

## Abstract

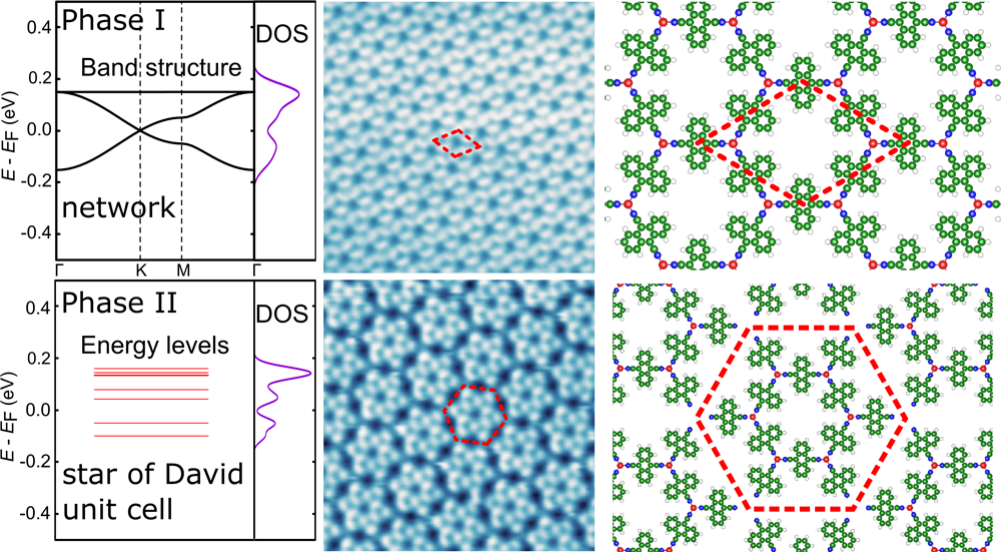

The combination of two-dimensional (2D) materials into vertical heterostructures has emerged as a promising path to designer quantum materials with exotic properties. Here, we extend this concept from inorganic 2D materials to 2D metal–organic frameworks (MOFs) that offer additional flexibility in realizing designer heterostructures. We successfully fabricate a monolayer 2D Cu-dicyanoanthracene MOF on a 2D van der Waals NbSe_2_ superconducting substrate. The structural and electronic properties of two different phases of the 2D MOF are characterized by low-temperature scanning tunneling microscopy (STM) and spectroscopy (STS), complemented by density-functional theory (DFT) calculations. These experiments allow us to follow the formation of the kagome band structure from Star of David-shaped building blocks. This work extends the synthesis and electronic tunability of 2D MOFs beyond the electronically less relevant metal and semiconducting surfaces to superconducting substrates, which are needed for the development of emerging quantum materials such as topological superconductors.

## Introduction

Two-dimensional (2D) materials have attracted broad attention because of their outstanding properties and wide range of material properties that can be realized.^1,2^ The properties of the individual materials can be further developed in van der Waals (vdW) heterostructures, exploiting the interactions between layers to fabricate designer systems based on exotic electronic properties. This is exemplified by twisted bilayer graphene samples exhibiting superconductivity and correlated insulator states.^3,4^ There are also examples of using vdW materials to realize exciton condensates, quantum spin liquids, Chern insulators, and topological superconductivity in vdW heterostructures.^3,5−9^

While recent research in realizing vdW heterostructures has focused on inorganic 2D materials, 2D metal–organic frameworks (MOF) form an extremely interesting, broad, and tunable class of materials. MOFs are well-established in topics such as single-atom catalysis or gas storage, but there is also growing interest in the intrinsic electronic properties of 2D MOFs. Theoretical works have predicted their usage to realize, for example, 2D topological insulators,^10−12^ half-metallic ferromagnetism,^13−16^ and quantum spin liquids.^17,18^

2D MOFs have been synthesized on metal surfaces by following the concepts of supramolecular coordination chemistry.^19,20^ So far, most of the 2D MOFs are made on coinage metal surfaces,^21−25^ where the interaction with the metal substrate strongly masks the intrinsic electronic properties of the MOF. This can be overcome by using weakly interacting substrates (such as graphene and hBN) that allow probing of the intrinsic exotic electronic properties of 2D MOFs.^26−31^ However, to realize the exciting prospect of truly designer materials, it is important to demonstrate MOF synthesis on other 2D substrates.

Among possible candidates of MOF-related designer materials, MOFs on superconductors are particularly interesting. Magnetic adsorbates on a superconducting surface give rise to the Yu–Shiba–Rusinov (YSR) states.^32−35^ Furthermore, 2D magnetic lattices on a superconductor (YSR lattice) can lead to intriguing 2D topological superconductivity.^9,36−40^ The YSR states have been observed on individual 3*d* transition metal-phthalocyanine molecules on superconducting substrates, including layered vdW material NbSe_2_.^35,41−44^ While MOFs have not been realized on superconducting transition metal dichalcogenides, Ahmadi et al. have fabricated Pb-TNAP and Na-TNAP networks on a Pb surface.^45^ However, the insufficient mobility of the adsorbates hinders the formation of an ordered transition metal-based MOF.^45^

In this work, we successfully fabricated a 2D Cu-dicyanoanthracene (DCA) MOF on a NbSe_2_ superconducting substrate under ultrahigh vacuum (UHV) conditions. The structural and electronic properties of the samples are studied by low-temperature scanning tunneling microscopy (STM) and spectroscopy (STS). The ordered DCA_3_Cu_2_ network shows a structure that is a combination of a honeycomb lattice of Cu atoms with a kagome lattice of DCA molecules. Interestingly, we observed an unexpected Star of David (SD) lattice phase after further annealing the sample at room temperature. The evolution of energy bands from one SD unit cell to the kagome band of 2D MOF was observed by comparing the STS of these two phases combined with density-functional theory (DFT) calculations. Given that a similar magnetic transition metal-based 2D MOF has been successfully fabricated on a weakly interacting graphene/Ir(11) substrate, we think that the same strategy can be easily applied to synthesis of 2D MOFs with magnetic transition atoms such as Co^27^ on a van der Waals superconductor—possibly leading to topological superconductivity.

## Results and Discussion

**Figure 1:**
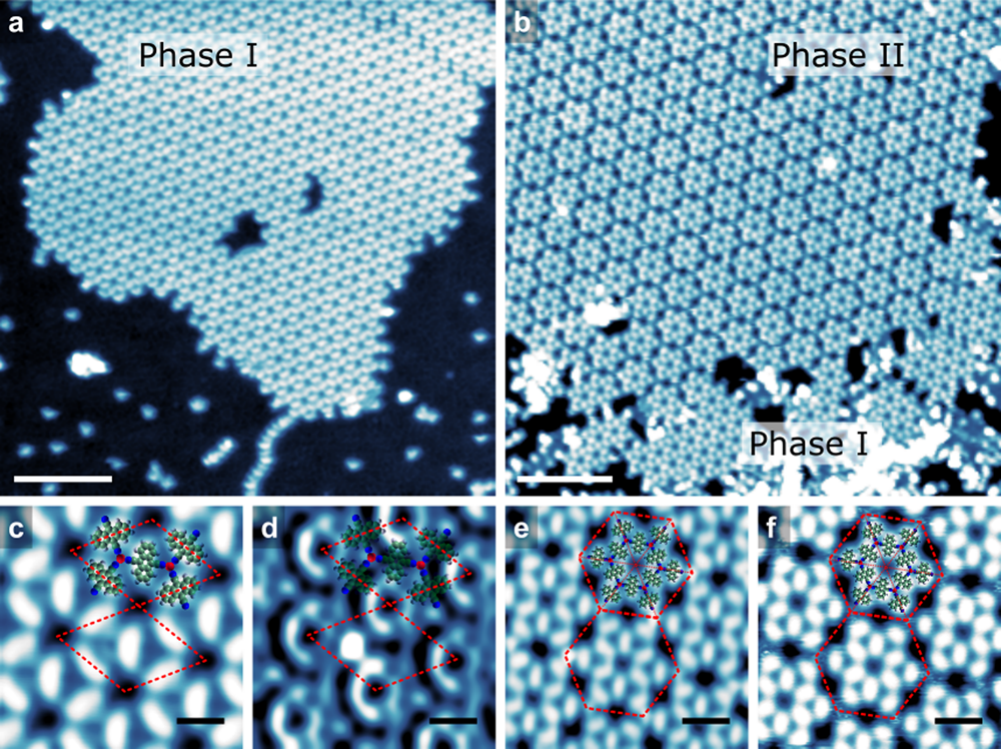
(a,b) STM overview images of Cu-DCA MOFs on the NbSe_2_ surface: (a) phase I, (b) phases I and II. (c,d) STM images of phase I. (e,f) STM images of phase II. The red parallelograms indicate the unit cells (C cyan, N blue, H white, Cu red). Imaging parameters: (a) 1.0 V and 2 pA, (b) 1.0 V and 10 pA, (c) 1.0 V and 11 pA, (d) 1.8 V and 11 pA, (e) 0.1 V and 10 pA, (f) 1.0 V and 10 pA. Scale bars: (a,b) 10, (c,d) 1, (e,f) 2 nm.

We deposited DCA molecules and Cu atoms sequentially onto the NbSe_2_ substrate held at room temperature (details given in the Methods section). In a stark contrast with the close-packed assembly of DCA molecules shown in Figure S1, an ordered DCA_3_Cu_2_ network (denoted as phase I) was observed in Figure 1a, where the Cu atoms arrange in a honeycomb lattice and the DCA molecules form a kagome pattern. Figures 1c and d show high-resolution images of a DCA_3_Cu_2_ network. Figure 1c shows the backbone of the network, with the unit cell shown as a red parallelogram with a lattice constant of *a* = 1.96 nm, which is in the range of previous reports of the same network on other substrates^30,46−49^ and comparable with the gas phase DFT values (2.02 nm). The electronic states of the DCA_3_Cu_2_ can be seen at a higher bias (1.8 V) shown in Figure 1d (see below for more detailed spectroscopy of the network electronic structure).

After further annealing the sample at room temperature, phase II emerged as the dominant pattern (see Figure 1b), where the DCA molecules formed an SD lattice, indicating that phase I is metastable. Figures 1e and f are the high-resolution images of phase II. At 0.1 V (Figure 1e), it can be seen that there are bright dots between each SD in phase II, while at 1.0 V (Figure 1f) those areas become fuzzy. The SD unit cell shown as a red hexagon in Figure 1e has a lattice constant of *a* = 4.33 nm, which is 10% larger than double the lattice constant of phase I. Note that phase I undergoes a 5% lattice mismatch with the lattice constant of a 6 × 6 NbSe_2_, while the distance of the centers of outer DCA molecules in a single cell of phase II (3.46 nm) almost perfectly matches the lattice constant of a 10 × 10 NbSe_2_ (0.5% mismatch) thus is more favorable after annealing. This appears to be coverage independent, as the experiments are always at submonolayer coverage.

**Figure 2:**
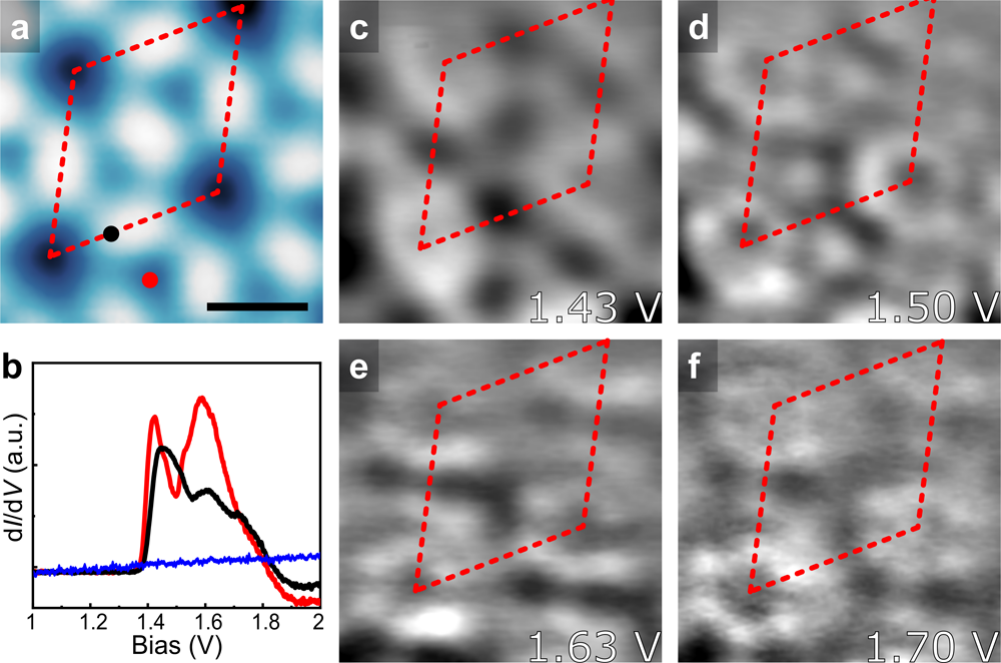
(a,b) STS (b) on phase I at the positions shown in part a. The blue curve in part b is a reference spectrum on bare NbSe_2_. (c–f) Experimentally recorded constant-height d*I*/d*V* maps at the energies indicated in the panels in the same area of part a. Imaging parameters: (a) 1.0 V and 10 pA. Scale bar: (a) 1 nm.

The d*I*/d*V* spectra recorded on a DCA molecule and a Cu atom in phase I are shown in Figures 2 and S2. Both spectra exhibit a broad feature in the energy range between 1.4 and 1.9 V. We recorded constant height d*I*/d*V* maps at representative biases indicated in Figure 2c–f, which show nearly uniform features at different biases. These characteristics are attributed to the band structure formed in the 2D MOF, which has been well studied in the same network on graphene/Ir(111).^30^ It can be seen that there is a region of negative differential conductance directly above the band of the MOF. This is not an intrinsic electronic feature of the 2D MOF but arises from the bias dependence of the tunneling barriers.^50−52^ It is worth noting that the energy position of this band shifts from close to the Fermi level on graphene/Ir(111) to 1.4 V on NbSe_2_. The energy band shift can be primarily explained by the different work functions (i.e., the energy difference between the vacuum level and the Fermi energy) of graphene/Ir(111) (4.65 ± 0.10 eV)^53^ and NbSe_2_ (5.9 eV).^54^ Due to the instability of the tunneling junction at negative bias, we were only able to get reliable STS results at biases above −0.5 V. We carried out DFT calculations considering phase I DCA_3_Cu_2_ on monolayer NbSe_2_, where the stacking geometries can be seen in Figure S3. Figure S4 shows that the band structure of the MOF in this heterostructure is 0.6 eV higher relative to the Fermi level, in qualitative agreement with our experimental findings.

**Figure 3:**
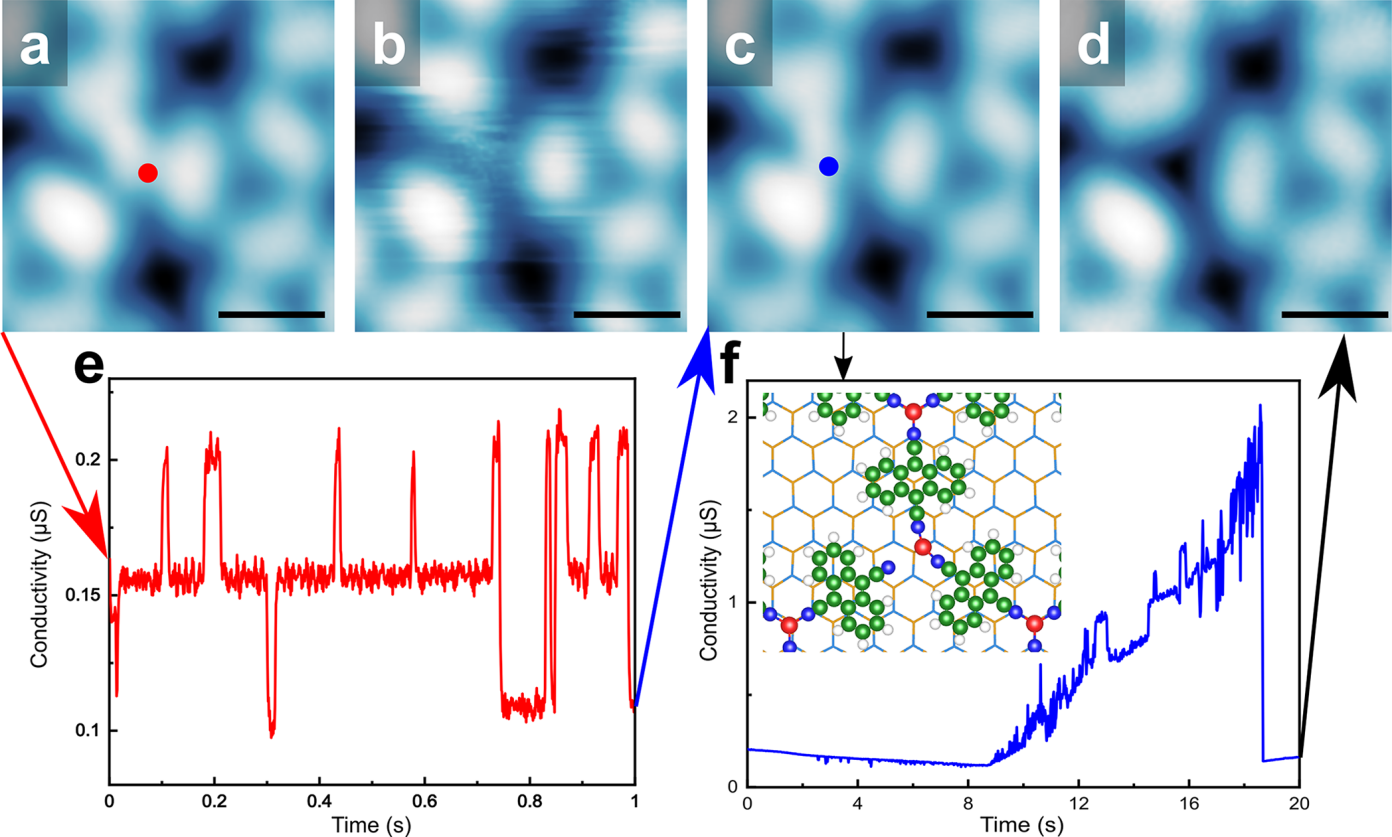
(a–d) STM images of phase II recorded before and after measuring the current versus time spectra. (e) Current versus time trace recorded at 0.4 V at the position indicated by the red dot. (f) Current versus time trace recorded at 3.0 V at the position indicated by the blue dot in part c. Imaging parameters: (a) 0.1 V and 10 pA, (b) 0.8 V and 10 pA, (c) 0.1 V and 10 pA, (d) 0.1 V and 10 pA. Scale bars: (a–d) 1 nm. (f inset) Outer Cu atom binding to two of the three N atoms of the correspondent DCA molecules (see also Figure S5), where the wireframe in the background represents the NbSe_2_ substrate.

The Cu–N bond is estimated to be 2.0 ± 0.2 Å within the SD, in line with our DFT result (1.9 Å) and with the value of the earlier report of Cu–N coordination bond.^55^ In contrast, the distance between the fuzzy center and the surrounding N atom is 3.5 ± 0.3 Å, which is beyond the range of a typical Cu–N bond. It can be seen from the images at low bias (0.1 V, Figures 3a and c) that the bright dot was not located at the center of the three surrounding molecules but only attached to two of the molecules. While at the bias of 0.8 V, the bright dot area became unstable and thus results in a fuzzy feature in Figure 3b, which also affects the surrounding DCA molecules. We measured the current versus time (*I*–*t*) traces at the positions indicated in Figures 3a and c. While using a relatively low bias (0.4 V) for the *I*–*t* spectrum, a switching between three current levels was clearly observed, as shown in Figure 3e. A plausible explanation would be the Cu atom hopping between the three possible binding geometries. When using a relatively high bias (3.0 V), the tunneling current became unstable and increased dramatically until a sharp drop occurred (Figure 3f). The sharp drop in current was likely because the Cu atom was picked up by the tip, which was further evidenced by the STM image taken after acquiring the *I*–*t* trace (Figure 3d). The inset of Figure 3f shows a portion of the phase II DCA_3_Cu_2_ on a monolayer NbSe_2_ obtained through our DFT calculations, which shows that the outer Cu atom of the SD will only bond to two N atoms in this scenario (see also Figure S5), which agrees with our experimental findings. In this case, the shortest distance measured between the Cu atom and the three surrounding N atoms is 1.9 Å, agreeing well with the Cu–N typical bond length, while the largest distance measured is 3.7 Å, again matching experiments.

**Figure 4:**
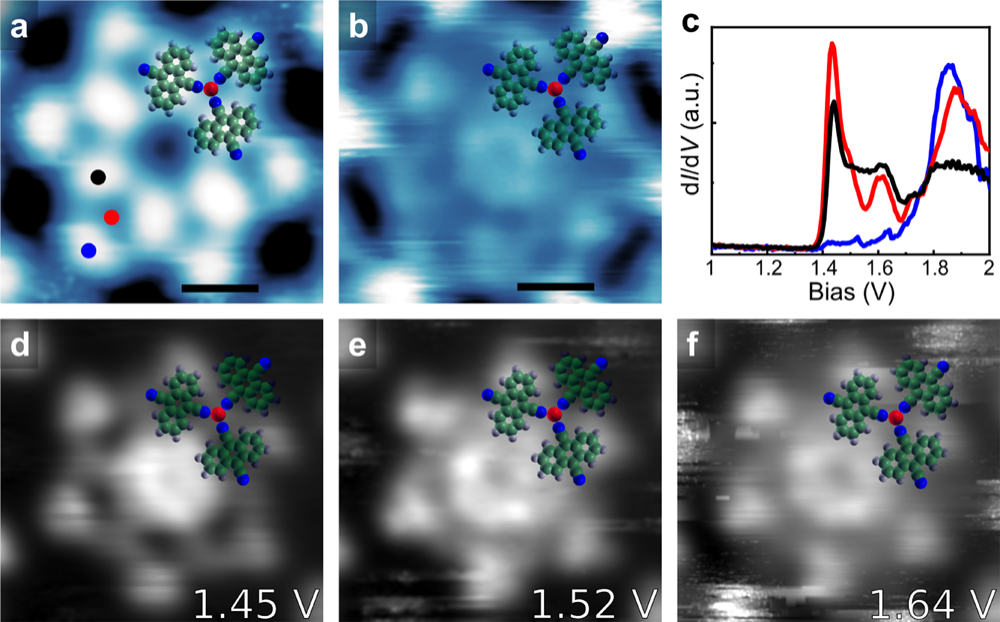
(a–c) STS recorded (c) on phase II at the positions shown in part a. (d–f) Experimentally recorded constant-height d*I*/d*V* maps at the energies indicated in the panels in the same area of parts a and b. Imaging parameters: (a) 1.0 V and 10 pA, (b) 2.0 V and 10 pA. Scale bars: (a,b) 1 nm.

The electronic properties of phase II are shown in Figures 4 and S6. While the d*I*/d*V* spectra recorded on the Cu atom and the inner DCA molecule still show the same broad feature similar to phase I with two peaks at 1.45 and 1.64 V, the outer DCA molecule has a very limited local density of states (LDOS) at these energies but shows a strong peak at 1.85 V, which is reflected on the neighboring Cu atom (the nearby CN group of the outer DCA molecule) as well. The constant height d*I*/d*V* maps below 1.6 V (Figures 4d and e) show LDOS intensity only on the inner DCA molecules and Cu atoms. At 1.64 V, while the character of the inner parts remains the same in the constant height d*I*/d*V* map (Figures 4f), the outer part becomes fuzzy due to Cu atom instability, as discussed above. An STM image recorded at 2.0 V (Figures 4b) shows the electronic contributions consisting of all the states from 0 to 2 V of phase II, where contrast within the SD is dominated by the ends of the long axis of the DCA molecules.

**Figure 5:**
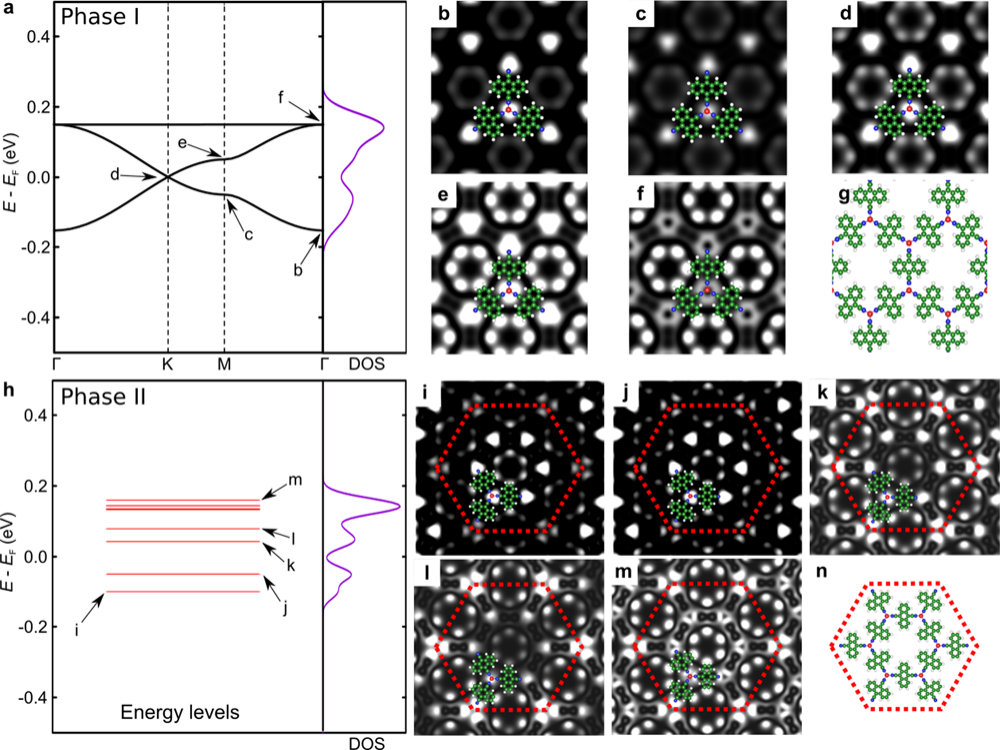
Calculated band structure, energy levels, DOS, simulated LDOS maps at the energies indicated in the band structure, and the related model of gas-phase Cu-DCA MOF. (upper panels) Phase I. (lower panels) Phase II. The red dashed lines in the lower panels indicate the SD unit cell of phase II.

The differences in the electronic properties between phases I and II can also be observed in their gas-phase state, as shown in our DFT simulations. Figure 5a shows the calculated band structure of the pristine, ordered DCA_3_Cu_2_ network (phase I), revealing a kagome band structure which consists of a Dirac band with an additional flat band pinned to the top of the Dirac band.^56−58^ We simulated the constant-height d*I*/d*V* maps by extracting the local density of states (LDOS) maps on selected points of the kagome band structure, as can be seen in Figures 5b–g. Overall, the simulations are consistent with our experimental findings. The LDOS maps in Figures 5b–d show that higher contrast is observed on the N atoms and the end of the axis of the DCA molecules, with a minor contribution from the Cu atoms (see also Figure S4), where the LDOS maps were extracted from energies within the Dirac bandlike spectrum of the kagome band. At higher energies closer to the flat band (Figures 5e,f), the LDOS contrast on the DCA molecules is significantly increased, which is consistent with the flat band arising from the entities located at the kagome sites of the unit cell.^30^ Note again that the kagome band is observed around the Fermi level in the gas-phase calculation, whereas in the experiment these features are observed 1.4 eV above the Fermi level. We stress here that calculations considering the NbSe_2_ substrate also give these features well above the Fermi level (0.6 eV), which agrees qualitatively with the experiment. However, the fact that the gas-phase calculation accurately captures the electronic properties of the DCA_3_Cu_2_ MOF shows that the layers (MOF and NbSe_2_) do not interact strongly with each other and retain their intrinsic properties in the heterostructure.

For phase II, as a simple approximation to simulate its electronic properties, we considered a periodic SD lattice fixing the experimental lattice parameter (4.33 nm) without the outer Cu atoms. Therefore, the distance between the SD units is large enough to isolate them, resulting in molecularlike energy levels rather than a dispersive band structure, such as for phase I. Nonetheless, the DOS of phase II shows similar characteristics to phase I since the lattice hosts a complete SD unit cell. The simulated LDOS maps of phase II at low bias (Figures 5i and j) show higher contrast on the inner entities, while at high bias (Figures 5k–m) the outer DCA molecules (especially the outer N atoms) possess stronger LDOS. These characteristics are in agreement with the experiment, where the outer DCA molecules have stronger DOS at higher bias (Figures 4c). In other words, the comparison between the two different phases allows us to visualize the evolution of energy bands from one SD unit cell to the kagome band of a 2D MOF.

## Conclusions

In summary, we have studied the structural and electronic properties of two different phases of 2D Cu-DCA MOF on a NbSe_2_ substrate under UHV conditions using experimental (STM/STS) and theoretical (DFT) methods. Phase I is an ordered DCA_3_Cu_2_ network possessing a kagome band structure. Phase II consists of a lattice of an electronically isolated Star of David (SD) network with unstable Cu atoms between them. The differences in the electronic structures of the two phases can be understood in terms of an evolution of the energy bands from one SD unit cell to the kagome band of 2D MOF. This work demonstrates the successful synthesis of a 2D MOF on the superconducting NbSe_2_ substrate capable of producing 2D MOF–superconductor hybrids with the proposed magnetic^27^ or organic topological insulator MOFs.^31,59−61^

## Methods

### Experimental Section

Sample preparation and STM experiments were carried out in an ultrahigh vacuum system with a base pressure of ∼ 10^−10^ mbar. The 2H–NbSe_2_ single crystal (HQ Graphene, The Netherlands) was cleaved *in situ* in the vacuum.^42^ The DCA_3_Cu_2_ network was fabricated by the sequential deposition of 9,10-dicyanoanthracene (DCA, Sigma-Aldrich) molecules (evaporation temperature 100 °C) and Cu atoms onto the NbSe_2_ substrate held at room temperature. The star phase emerged after annealing 10 h at room temperature or 5 min at 40 °C. Subsequently, the samples were inserted into the low-temperature STM (Createc GmbH), and all subsequent experiments were performed at *T* = 5 K. STM images were recorded in constant current mode. d*I*/d*V* spectra were recorded by standard lock-in detection while sweeping the sample bias in an open feedback loop configuration, with a peak-to-peak bias modulation of 15–20 mV at a frequency of 526 Hz. The STM images were processed with Gwyddion software.^62^

### Computational

DFT calculations were performed using the FHI-AIMS code.^63^ The default calculation setup used a “tight” basis set and the Perdew–Burke–Ernzerhof (PBE) exchange-correlation functional^64^ augmented with van der Waals terms through the Tkatchenko–Scheffler method.^65^ Within this methodology, different strategies were applied for the gas phase and on surface calculations, which are described as follows.

#### Gas Phase

For phase I DCA_3_Cu_2_, the geometry was fully relaxed considering a 2 × 2 × 1 uniform *k*-grid, while for phase II we kept the experimental lattice parameter fixed and a single Γ *k*-point was used. Electronic properties such as band structures, energy levels, density of states, as well as the LDOS maps were obtained by partially including exact exchange terms into the exchange-correlation functional by means of the hybrid Heyd–Scuseria–Ernzerhof (HSE06) functional,^66−68^ which has been shown to be relevant for describing the electronic properties of MOFs.^10,69^ A significantly larger *k*-grid was used for the band structures and density of states calculations. LDOS maps were computed by means of the PP-STM code,^70^ where the broadening parameter η was set to 0.2 eV.

#### On Surface

For the phase I DCA_3_Cu_2_ on NbSe_2_, the DCA_3_Cu_2_ was freely relaxed on a fixed 6 × 6 supercell NbSe_2_ monolayer with a 2 × 2 × 1 uniform *k*-grid. A strain of 3% was present on phase I DCA_3_Cu_2_ due to the mismatch between its lattice parameter and NbSe_2_. The hybrid HSE06 functional was used to obtain the electronic properties. For phase II DCA_3_Cu_2_ on NbSe_2_, the DCA_3_Cu_2_ was freely relaxed on a fixed 12 × 12 supercell NbSe_2_ monolayer.

In this case we used the “light” basis set due to the large size of the system. Figures S3 and S5 show the unit cell, atomic structures, and stacking configuration of all structures considered here.

## Supporting Information Available

STM image of close-packed assembly of DCA molecules on NbSe_2_; long-range STS recorded on phases I and II; calculation results of phases I and II on NbSe_2_ (PDF)
