# Supplementary material for: Two-Dimensional Metal–Organic Framework on Superconducting NbSe2
Source: ACS Nano. 2021 Nov 3;15(11):17813–9. doi: 10.1021/acsnano.1c05986 (PMC8613900; doi:10.1021/acsnano.1c05986)
Supplement: Supplementary file 1 — nn1c05986_si_001.pdf [file nn1c05986_si_001.pdf]

Supporting Information: A Two-Dimensional Metal-Organic Framework on Superconducting $NbSe_2$

Linghao Yan,[*,†,¶] Orlando J. Silveira,[†,¶] Benjamin Alldritt,[†] Shawulienu Kezilebieke,[†] Adam S. Foster,[†] and Peter Liljeroth[*,†]

†*Department of Applied Physics, Aalto University, FI-00076 Aalto, Finland*

‡*Nano Life Science Institute (WPI-NanoLSI), Kanazawa University, Kakuma-machi, Kanazawa 920-1192, Japan*

¶*Contributed equally to this work*

E-mail: linghao.yan@aalto.fi; peter.liljeroth@aalto.fi

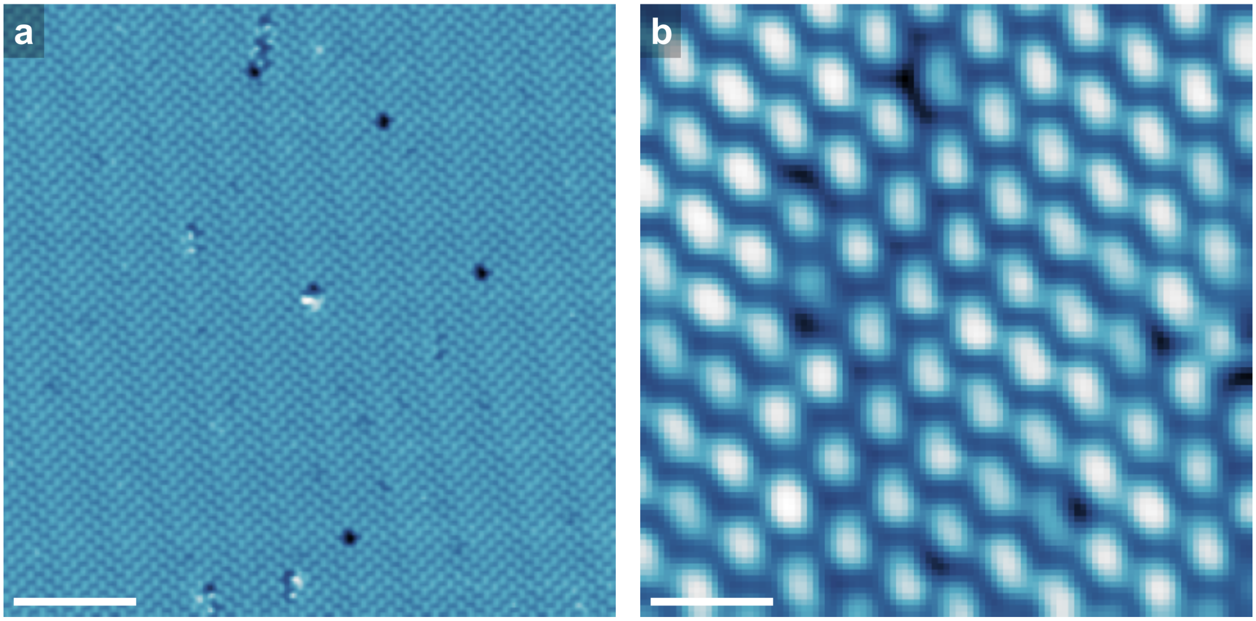

Figure S1: Close-packed assembly of DCA molecules achieved by depositing it on $NbSe_2$ substrate kept at room temperature. Imaging parameters: (a) 1.0 V and 10 pA, (b) -1.0 V and 10 pA. Scale bars: (a) 10 nm, (b) 2 nm.

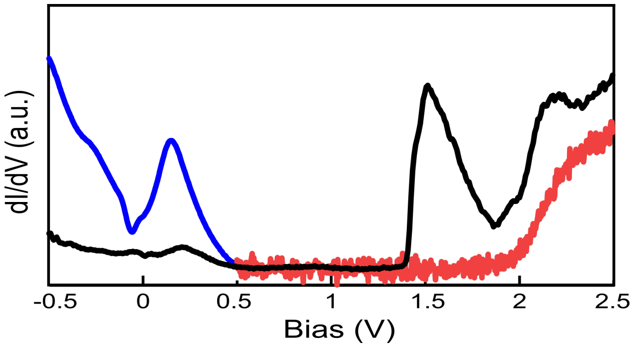

Figure S2: Long-range STS recorded on phase I (black) and on bare $NbSe_2$ substrate (red and blue). In order to observe the DOS of bare $NbSe_2$ at low bias ranges clearly, the spectra are taken separately in two parts (red and blue). By comparing the spectra, it can be seen that the DOS in the energy range between 1.4 V to 1.9 V are from the Cu-DCA MOF itself.

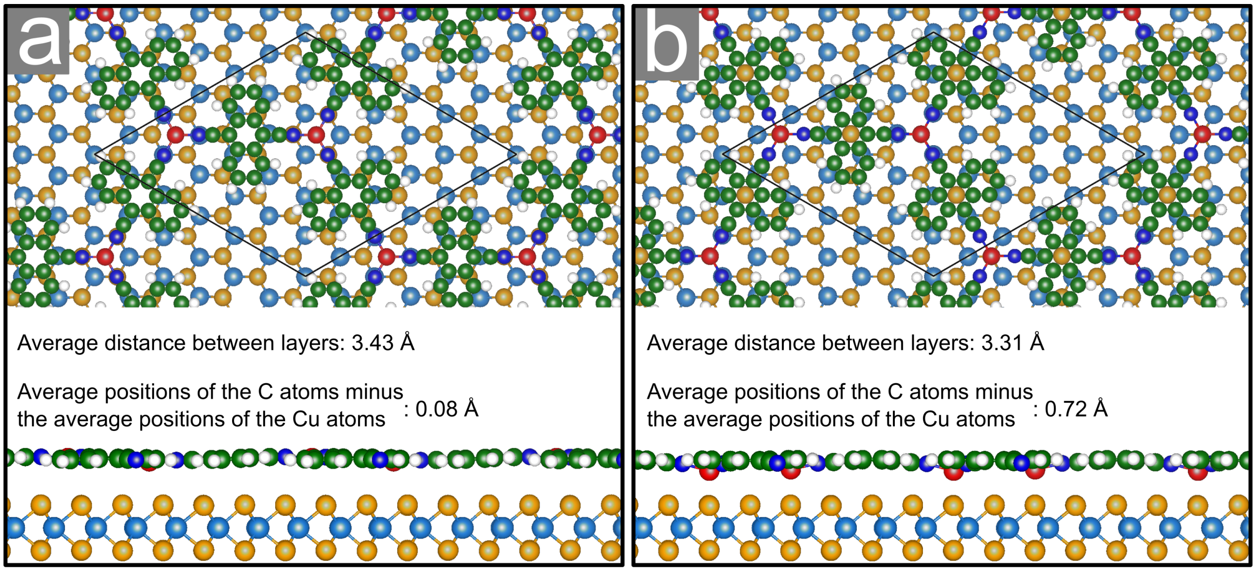

Figure S3: Stacking configurations obtained by relaxing the DCA_2Cu_3 phase I on a fixed $NbSe_2$ layer. In (a) both Cu atoms of the DCA_2Cu_3 are located above the hollow site of the $NbSe_2$ layer, while in (b) both Cu atoms are located above the Se atoms. For both cases the stacking geometry obtained after relaxation was the same as the initial configuration considered. We also considered a third case where the Cu atoms were located above the Nb atoms, but the final configuration was the same as (b) after structural optimization. The most energetically stable configuration is the one shown in (b), where the total energy is 0.61 eV smaller than in (a). The $NbSe_2$ layer is a 6×6 supercell constructed by doubling the 3×3 supercell of the $NbSe_2$ in the CDW phase, and the initial configurations of the heterostructure were built by putting the DCA_2Cu_3 phase I 3 Å above the top atomic layer of the $NbSe_2$. The lattice parameter of 20.87 Å was also kept fixed during relation, which leads to a small strain of 3% on the pristine DCA_2Cu_3 phase I layer.

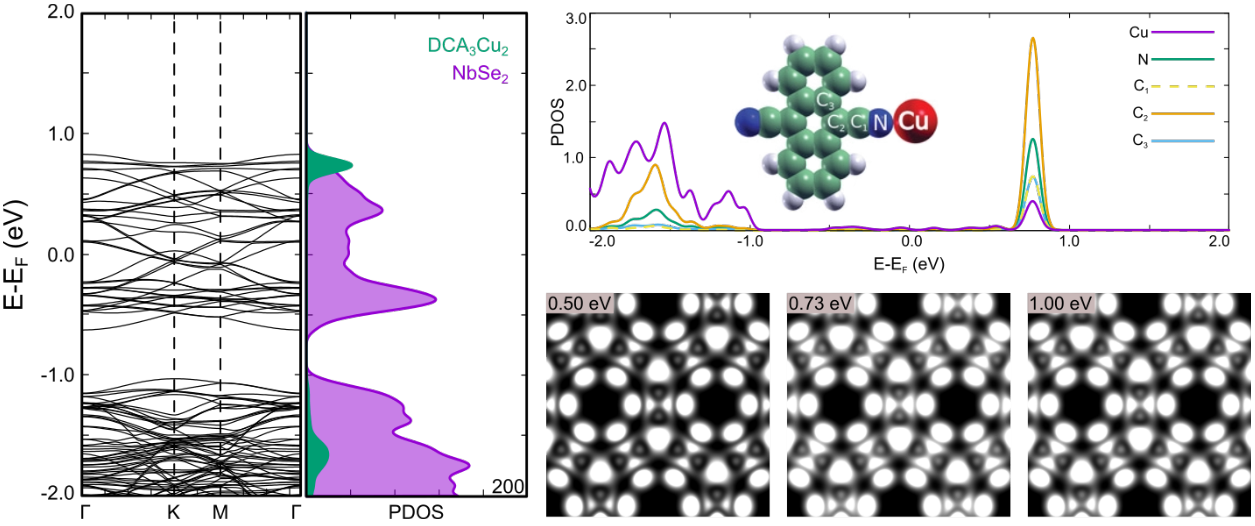

Figure S4: Left panel: Band structure and normalized projected density of states (PDOS) of the $DCA_3Cu_2/NbSe_2$ most favorable heterostructure. Right panel: PDOS of selected atoms of the DCA molecule in the $DCA_3Cu_2/NbSe_2$ heterostructure and LDOS maps. The energy levels in which the LDOS maps were extracted are indicated in the figures.

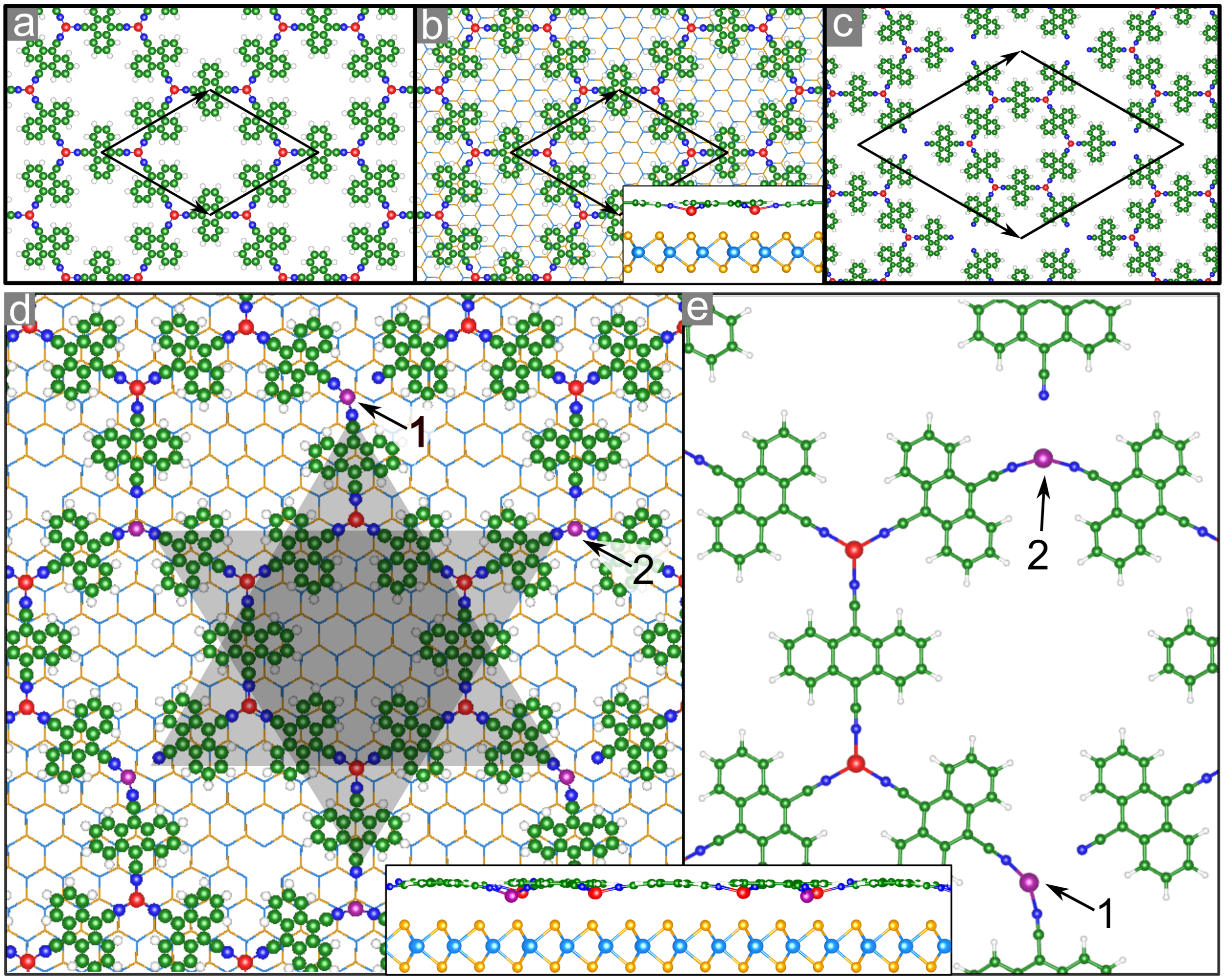

Figure S5: (a) and (b) are the geometries of the DCA_3Cu_2 gas phase, phase I, and the most favorable stacking geometry of the DCA_3Cu_2 phase I on top of the $NbSe_2$ monolayer, respectively. (c) and (d) are the geometries of the DCA_3Cu_2 gas phase, phase II, and of the DCA_3Cu_2 phase II on top of the $NbSe_2$ monolayer, respectively. In both (b) and (d) the top view of the geometries are shown, where the $NbSe_2$ is shown in a wireframe fashion, while the side views of the respective structures are shown as insets. In (d) the Cu atoms that bond to only two molecules are shown in a slightly different color, and (e) shows with more details these Cu atoms, where the $NbSe_2$ monolayer was removed for a clearer view.

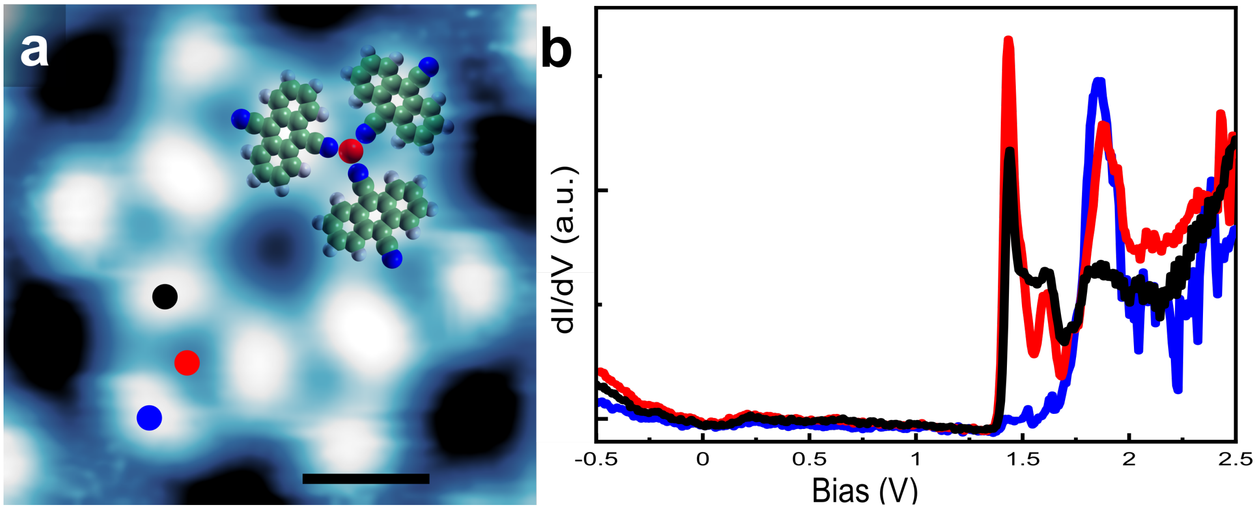

Figure S6: Long-range STS (b) on phase II at the positions shown in (a). Imaging parameters: (a) 1.0 V and 10 pA. Scale bar of (a): 1 nm.
